# An overview of artificial intelligence in diabetic retinopathy and other ocular diseases

**DOI:** 10.3389/fpubh.2022.971943

**Published:** 2022-10-28

**Authors:** Bin Sheng, Xiaosi Chen, Tingyao Li, Tianxing Ma, Yang Yang, Lei Bi, Xinyuan Zhang

**Affiliations:** ^1^Department of Computer Science and Engineering, Shanghai Jiao Tong University, Shanghai, China; ^2^Beijing Retinal and Choroidal Vascular Diseases Study Group, Beijing Tongren Hospital, Beijing, China; ^3^Beijing Tongren Eye Center, Beijing Tongren Hospital, Capital Medical University, Beijing, China; ^4^Chongqing University-University of Cincinnati Joint Co-op Institute, Chongqing University, Chongqing, China; ^5^School of Computer Science, University of Sydney, Sydney, NSW, Australia

**Keywords:** artificial intelligence, diabetic retinopathy, glaucoma, cataract, age-related macular degeneration

## Abstract

Artificial intelligence (AI), also known as machine intelligence, is a branch of science that empowers machines using human intelligence. AI refers to the technology of rendering human intelligence through computer programs. From healthcare to the precise prevention, diagnosis, and management of diseases, AI is progressing rapidly in various interdisciplinary fields, including ophthalmology. Ophthalmology is at the forefront of AI in medicine because the diagnosis of ocular diseases heavy reliance on imaging. Recently, deep learning-based AI screening and prediction models have been applied to the most common visual impairment and blindness diseases, including glaucoma, cataract, age-related macular degeneration (ARMD), and diabetic retinopathy (DR). The success of AI in medicine is primarily attributed to the development of deep learning algorithms, which are computational models composed of multiple layers of simulated neurons. These models can learn the representations of data at multiple levels of abstraction. The Inception-v3 algorithm and transfer learning concept have been applied in DR and ARMD to reuse fundus image features learned from natural images (non-medical images) to train an AI system with a fraction of the commonly used training data (<1%). The trained AI system achieved performance comparable to that of human experts in classifying ARMD and diabetic macular edema on optical coherence tomography images. In this study, we highlight the fundamental concepts of AI and its application in these four major ocular diseases and further discuss the current challenges, as well as the prospects in ophthalmology.

## Introduction

Artificial intelligence (AI) is a broad branch of computer science that develops theories, methods, technologies, and application systems to simulate, extend, and expand human intelligence in machines (1). Machine learning (ML) (2) is a subcategory of AI that uses statistical techniques to build intelligent systems. Using either a supervised or unsupervised approach, the intelligent system can learn and improve its performance automatically, such as accuracy, without being explicitly programmed. Deep learning (DL) (3), which uses advanced ML techniques, has achieved great success in computer vision and natural language processing tasks. This success is primarily attributed to its excellent feature extraction and pattern recognition capabilities, which use multiple processing layers (artificial neurons) to learn representations of data with different levels of abstraction (4) such that it associates the input with a diagnostic output. Because of this outstanding success, many investigators have applied DL to medical and healthcare-related tasks, such that DL has become a powerful tool in intelligent screening, diagnosis, and treatment of various diseases recently. DL has been used for COVID-19 detection from chest X-rays (5), thyroid classification from ultrasound imaging (6, 7), and lung nodule detection and staging from computed tomography (CT) images (8, 9).

Currently, AI has achieved radiologist-level diagnosis of medical images by learning from example images, which has significantly improved clinical workflows. The application of AI for medical image analysis plays an important role in maximizing efficiency and enhancing the accuracy of diagnosis and treatment for physicians. AI application also plays a significant role in improving current logistic and economic issues, which could influence the healthcare system by expanding clinical capacity and augments. Furthermore, AI is useful as an important auxiliary tool in the early detection of diseases, particularly in low-resource clinical settings. Based on fundus photographs and optical coherence tomography (OCT), in the field of ophthalmology, DL has been applied to four major eye diseases that cause blindness, including diabetic retinopathy (DR) (10–13), glaucoma (13, 14), age-related macular degeneration (ARMD) (13, 15, 16), and cataracts (17). AI has shown great promise in the auxiliary diagnosis of refractive error (18), retinopathy of prematurity (ROP) (19), retinal detachment (20), choroidal disease (21), and ocular tumors (22). Early detection is particularly crucial to prevent delays in treatment and vision loss.

AI simulates the thinking and diagnostic capabilities of doctors by learning their expertise and medical data to provide efficient and accurate diagnoses and personalized treatment plans in a short period using medical images and other relevant data. The IBM Watson System, a question-answering system, can effectively provide diagnostic and treatment strategies for patients with lung, prostate, and other cancers. This system was successfully developed by learning from empirical evidence-based medical articles, publications, treatment plans, clinical data, and experimental reports.

Personal health data in the future can be dynamically monitored through wearable devices and smart home devices, which will provide a wealth of data for medical diagnosis. Modeling with these personal health data will allow accurate personal health information to predict disease risk in a standardized and accurate manner. Artificial intelligence provides accurate guidance on the management of blood glucose and blood pressure, serves as a medication reminder, monitors health elements, and offers the population with comprehensive, full-cycle health services in a high-quality, intelligent, and daily manner.

The recent development of AI algorithms is providing unprecedented opportunities to address some major challenges in DR and other ocular diseases. For instance, the Inception-v3 algorithm trained with annotated fundus images can achieve diagnosis performance comparable with human experts. Although there exist several related reviews in the community, the technical background, unfortunately, has not been thoroughly investigated. In this study, we highlight the fundamental concepts of AI and its application in four major ocular diseases, and further discuss the current challenges, as well as the prospects in ophthalmology, providing unexplored insight in this area. The ability to introduce the fundamental concept of AI with reference to its clinical applications will increase the awareness of using AI in the community and discover new capabilities in the analysis of ocular diseases.

## Method of literature research

In this overview, we retrieved English articles from the commonly used database engines, including Pubmed/MEDLINE, Springerlink, the Cochrane Library, Google Scholar, and EMbase Medline with the keywords “Artificial Intelligence,” “Machine Learning,” “Deep Learning,” combined with keywords, including “diabetic retinopathy,” “cataract,” “glaucoma,” and “age-related macular degeneration.” The end date for the retrieval is December 2021. Studies retrieved by each pair of keywords were then combined to build an objective dataset of articles. A comprehensive review by several authors was performed of all references cited in the dataset. Proposals protocols, reviews, letters, opinions, and studies and/or articles that were not peer-reviewed were excluded. Publications relevant to our topic were selected and are found in the references. In this study, we focused on giving an overview of the application of AI in DR and other ocular diseases. We, therefore, attempt to select representative AI techniques for each disease category. We acknowledge that not all the articles under these keywords' combinations were included for discussion, providing more of a perspective and opinion review.

## AI's impact on human ocular diseases

### Diabetic retinopathy

DR is a leading cause of blindness in working populations in both developed and developing countries and is the most serious eye complication of diabetes mellitus (DM). The International Diabetes Federation estimates that by 2040, approximately 600 million people worldwide will have DM, one-third of whom will eventually develop DR. According to a meta-analysis consisting of 35 cohort studies with 22,869 subjects, the global prevalence of DR is 34.6% and vision-threatening DR is 10.2%, accounting for 51% of blindness cases worldwide (23).

**Figure 1:**
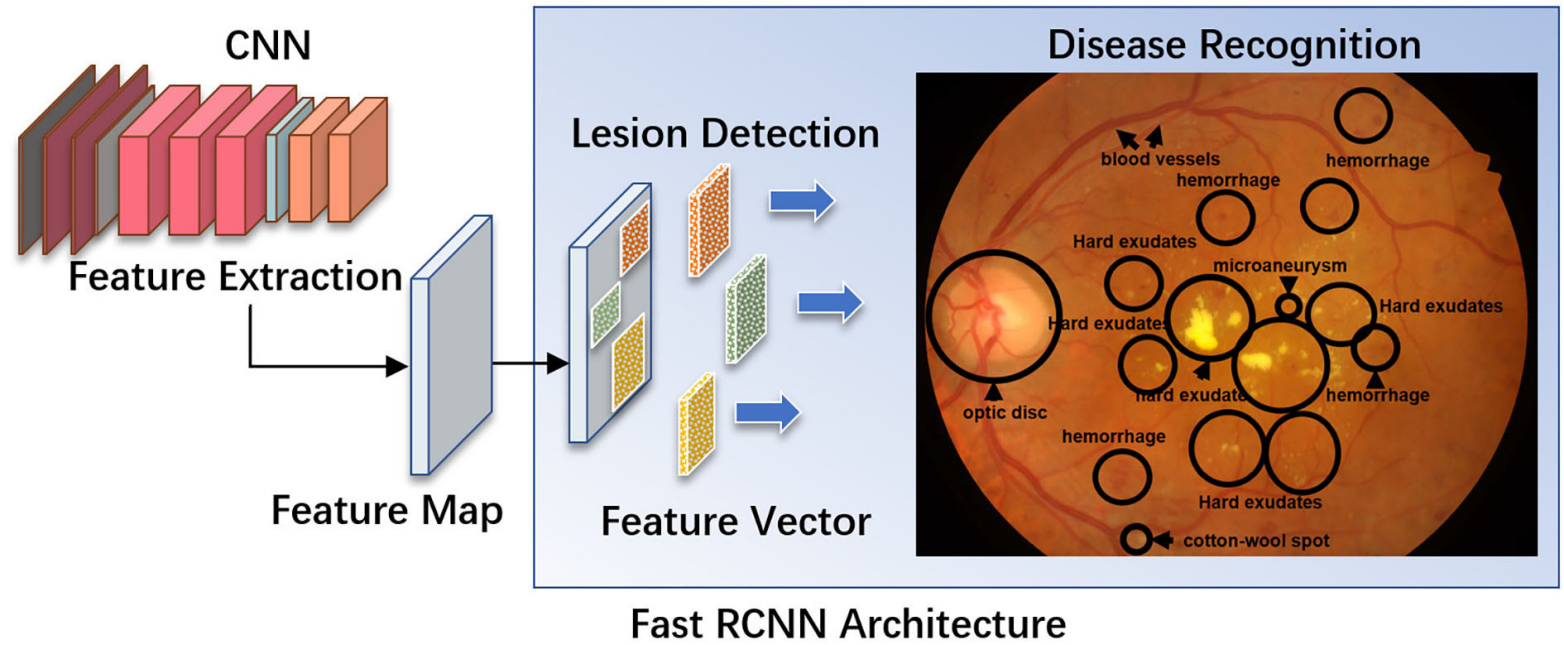
A diagram illustrating a fast R-CNN algorithm for automatic lesion detection and disease recognition from fundus images. The input fundus image will be fed into the CNN network to get the corresponding feature map. The derived feature map will be used to estimate region proposals (candidate lesion regions in squared boxes), which will then be classified and predicted as different disease categories. FC, fully connected layers; DM, diabetes mellitus; DR, diabetic retinopathy; R-CNN, region-based convolutional neural network; ROI, region of interest.

Regular DR screening is important for the timely treatment and prevention of vision loss (24). Time and financial constraints are major issues for both ophthalmologists and endocrinologists. The effectiveness of fundus photograph-based screening is significantly impacted by the limited number of registered ophthalmologists, particularly retinal specialists. DR is the most common retinal vascular disease, with typical fundus characteristics, including microaneurysms, hemorrhages, exudates, and neovascularization. For automatic screening of disease, these lesions must first be manually labeled on fundus images, and then a preliminary diagnosis using ML is made (Figure 1). In April 2018, the U.S. Food and Drug Administration (U.S. FDA) approved the first AI-assisted DR detection device, IDx-DR, for primary eye care, to aid physicians in DR screening (25, 26).

Compared to humans, ML can detect DR in a faster and more accurate manner (27). Furthermore, deep neural networks offer significantly higher predictive performance for DR screening using retinal images (28, 29). The AI-based DR screening model is feasible and acceptable for patients in endocrinology outpatient settings (30).

The performance of DL models relies heavily on the availability of sufficient training datasets. However, owing to complicated data acquisition procedures and ethical constraints, it is challenging to acquire sufficient data in the real world. To overcome this limitation, investigators have used migration learning to train a neural network with a small fraction of data and have used features from conventional methods. This method provides comparable performance to human experts in the classification of ARMD and diabetic macular edema (DME) (31). Other researchers have developed a self-supervised training scheme to train neural networks with many unlabeled medical images (32).

Diabetic choroidal vasculopathy (DCV) has been a hot research topic recently. Early detection of DCV could offer warning information regarding the occurrence of DR in patients with DR. However, automatic segmentation of the choroidal layer remains a challenging task because of the low contrast, inhomogeneous intensity, inconsistent texture, and blurred boundaries between the choroid and sclera in the OCT images. Currently used methods continue to emphasize manually or semi-automatically segmenting areas of interest. The researchers proposed segmenting the Bruch's membrane (BM) in OCT images using a series of morphological operations, while the choroidal layer was segmented using a DL approach (21). Moreover, a segmentation method based on the adaptive appearance and prior shape information was developed to separate the retinal layers (33).

Recently, DL systems for detecting DR have developed rapidly, with remarkable results (12, 13, 26, 34), greatly improving the diagnostic performance of non-proliferative DR (NPDR) and middle- and late-stage PDR (Table 1). Researchers have also extended their research to the grading and prediction of DR based on lesion identification. The International Clinical Classification of Referable DR (rDR) defines DR as moderate, severe non-proliferative DR (NPDR), proliferative DR (PDR), and/or macular edema (ME). Abràmoff et al. showed that AlexNet and VGG achieved 96.8% sensitivity, 87% specificity, and 98% area under the curve (AUC), respectively (10). The team defined mild and beyond classification DR (mtmDR) as ETDRS grade 35 or higher, and/or DME in at least one eye, based on the early treatment diabetic retinopathy study severity scale (ETDRS) and diabetic macular edema (DME). This AI system had a sensitivity of 87.2%, a specificity of 90.7%, and an imageability of 96.1% (34). Gulshan et al. used CNN to classify the referable diabetic retinopathy (rDR) as moderate or worse diabetic retinopathy, referable diabetic macular edema, or both and achieved 97.05% sensitivity, 93.4% specificity, and 99.1% AUC (12). DL system from Google AI Healthcare identified image features to grade fundus lesions derived from 128,175 retinal images (labeled by 54 ophthalmologists) and discovered that these image features could quickly identify DR and identify signs of DR. Ting et al. reported a clinically acceptable diagnostic performance with an AUC of 93.6%, sensitivity of 90.5%, and specificity of 91.6%, in detecting DR using a development dataset acquired from Singapore integrated DR Program and several external datasets from six different countries (13). In another study, investigators from Aalto University trained a DL model based on Inception-v3 and found that DL could accurately separate DR and macular edema (35). Feng Li et al. optimized the Inception-v4 algorithm with a multiple-improvement depth ensemble to detect DR and DMO and achieved an AUC, sensitivity, and specificity of 99.2%, 92.5%, and 96.1% (36), respectively. Reguant et al. visualized the neural network decision process and analyzed image features; discovered that Inception-v3, recognition deep residual learning (ResNet) 50, InceptionresNet50, and Xception achieved 89–95% accuracy with AUC, sensitivity, and specificity of 95–98%, 74–86%, and 93–97%, respectively, for disease classification of DR (37). Ryu et al. proposed a convolutional neural network (CNN) model for diagnosing DR based on optical coherence tomography angiography (OCTA) images, achieving 91–98% accuracy, 86–97% sensitivity, 94–99% specificity, and 91.9–97.6% AUC (38).

**Table 1:** Typical deep learning systems for NPDR and PDR.

**References**	**Year**	**Modality**	**Diseases**	**Test set**	**Number of images in test set**	**CNN**	**AUC**	**Sensitivity (%)**	**Specificity (%)**
Abràmoff et al. (10)	2016	CFP	No DR, rDR, vtDR, ME	Messidor-2	1,748	AlexNet/VGG	0.98	96.8	87.0
Gulshan et al. (12)	2016	CFP	No DR, mild DR, moderate DR, severe DR, PDR, rDME, rDR	EyePACS-1	9,963	—	0.991	97.5	93.4
Ting et al. (13)	2017	CFP	DR, possible glaucoma, AMD	SiDRP 14-15	71,896	VGG-19	0.936	90.5	91.6
Abràmoff et al. (34)	2018	OCT	DR, DME	Data from 10 clinical centers in the United States	892 patients	AlexNet/VGG	—	87.2	90.7
Li et al. (36)	2021	CFP	DR, DMO	Messidor-2	8,739	Improved Inception-v4	0.992	92.5	96.1
Reguant et al. (37)	2021	CFP	No DR, mild NPDR, moderate NPDR, severe NPDR, PDR	EyePACS and DIARETDB1	35,122	CNNs	0.95–0.98	74–86	93–97
Ryu et al. (38)	2021	OCTA	DR	OCTA	240	ResNet101	0.919–0.976	86–97	94–99
Dai et al. (39)	2021	CFP	No DR, mild NPDR, moderate NPDR, severe NPDR, PDR, DME	NDSP/ EyePACS	27,948/88,702	DeepDR	0.944/0.943	—	—

AI, Artificial intelligence; AlexNet, Deep Convolutional Neural Networks; AMD, age-related macular degeneration; AUC, area under the curve; CFP, Color fundus photography; CNN, convolutional neural network; DIARETDB1, Standard Diabetic Retinopathy Database Calibration level 1; DMO, diabetic macular edema; EyePACS, Kaggle EyePACS dataset; SiDRP, Singapore Integrated Diabetic Retinopathy Program; ME, macular edema; Messidor, Methods to evaluate segmentation and indexing techniques in the field of retinal ophthalmology dataset; NDSP, Nicheng Diabetes Screening Project cohort; NPDR, Non-proliferative diabetic retinopathy; OCT, optical coherence tomography; OCTA, optical coherence tomography angiography; PDR, Proliferative diabetic retinopathy; rDME, referable diabetic macular edema, rDR, referable diabetic retinopathy; ResNet, recognition deep residual learning; VGG, Very Deep Convolutional Networks; vtDR, vision-threatening diabetic retinopathy.

Researchers from Shanghai Jiao Tong University proposed a deep neural network-based AI algorithm for detecting early DR and microaneurysms, which significantly improves the accuracy of the automatic detection of early DR and STDR, including proliferative DR and DME (39). A system for the automatic diagnosis of diabetic fundus lesions has been developed to assist in understanding the grading of fundus lesions and the severity of the disease in patients. The investigators have also developed a portable fundus photography device, which consists of a detector lens, smartphone, and fixed holder, allowing users to take fundus photographs anywhere. The fundus photographs obtained can be transmitted to a server for diagnostic analysis, including optic disc and macular localization, vascular segmentation, lesion detection, and lesion grading. The diagnostic results of this system were compared with those of ophthalmologists and achieved an accuracy rate of 85% (16). Furthermore, the researchers proposed an algorithm for optic disc and macular region detection based on a kernel least squares classifier. This algorithm uses several already labeled optic disc and macular region images to complete optic disc boundary localization and establish an accurate mapping from the image to the region. Based on this, the researchers constructed a method to accurately detect the optic disc region and locate the center of the optic disc for color retinal images, which is based on a kernel least-squares classifier to calculate the optic disc area. The method is then based on multimodal information to detect the site of vascular aggregation and obtain the optic disc center with higher accuracy. Particularly, in terms of optic disc localization, this method successfully detected 332 images out of 340 testing images, with a detection success rate of 97.65%. For optic disc boundary detection, the method achieved a success rate of 94.54% among all 112 images in the digital retinal images for vessel extraction (DRIVE) and structured analysis of the retina (STARE) databases; in macular area detection, 330 images were detected on all 340 test images, achieving a detection success rate of 97.06%. In the global finals of intelligent reading of fundus images at the 2018 IEEE International Symposium on Biomedical Imaging (ISBI), the optic disc detection and macular center detection technologies developed independently by researchers won first place worldwide (40). Furthermore, the detection and analysis of blood vessels in fundus images are crucial for the diagnosis of related diseases (41). Researchers have proposed an automatic extraction algorithm for blood vessels in fundus images based on direction-aware detectors, which constructs an orientation-aware detector that can accurately extract blood vessels from fundus images. The detector learns the orientation and distribution characteristics of blood vessels using the energy distribution of the Fourier transform and then extracts the blood vessel morphology using a dual-scale segmentation method, in which a linear operator is used for the large-scale, and a Gabor filter bank is used for the small-scale, making the detector more robust and structure-aware. According to the authoritative standard connectivity, area, and length (CAL) proposed by Gegndez-Arias, the algorithm achieved an accuracy of 80.82% on the international public dataset DRIVE and 68.94% on the STARE dataset. Experimental results show that the proposed method outperforms the existing segmentation methods and has high accuracy and robustness. Furthermore, investigators added a weakly supervised sensitive heat map (WSSH) to the CNN to create a CNN-WSSH model, combining the automatic detection of DR classification with a weakly supervised localization method to address the localization challenge (42).

DL methods enable regular screening in various locations, particularly in rural areas, making the early detection of common chronic diseases easier. To address the lack of medical resources, researchers have evaluated the role of automated AI-based software in DR and STDR, providing an initial tool for mass retinal screening for patients with diabetes using smartphone devices to take fundus photos and validate them against an ophthalmologist's score (43). Furthermore, fundus images acquired by patients using self-filming fundus imaging (SFI) are comparable in image quality to those acquired by trained specialists (44).

Through a prospective study of fundus images taken with smartphones, the researchers concluded that DL models are generalizable in identifying chronic kidney disease and type 2 diabetes, and feasible in predicting disease progression in a longitudinal cohort (45).

We anticipate that AI algorithms will improve their ability to predict the onset and progression of DR more effectively and concisely.

### Cataract

A cataract is a metabolic dysfunction disorder with variable pathological factors, such as aging, genetics, local nutritional disorders, immune and metabolic abnormalities, trauma, poisoning, and radiation, resulting in protein denaturation in the lens. Cataracts account for up to 18.4% of visual impairment and 33.4% of blindness worldwide (46). It is critical to screen people with diabetes for age-related cataracts to prevent blindness. Slit lamp examination and iris projection methods are mostly used in the examination of cataracts. However, compared with these two methods, the non-dilated fundus photography method has convenient and effective features.

**Figure 2:**
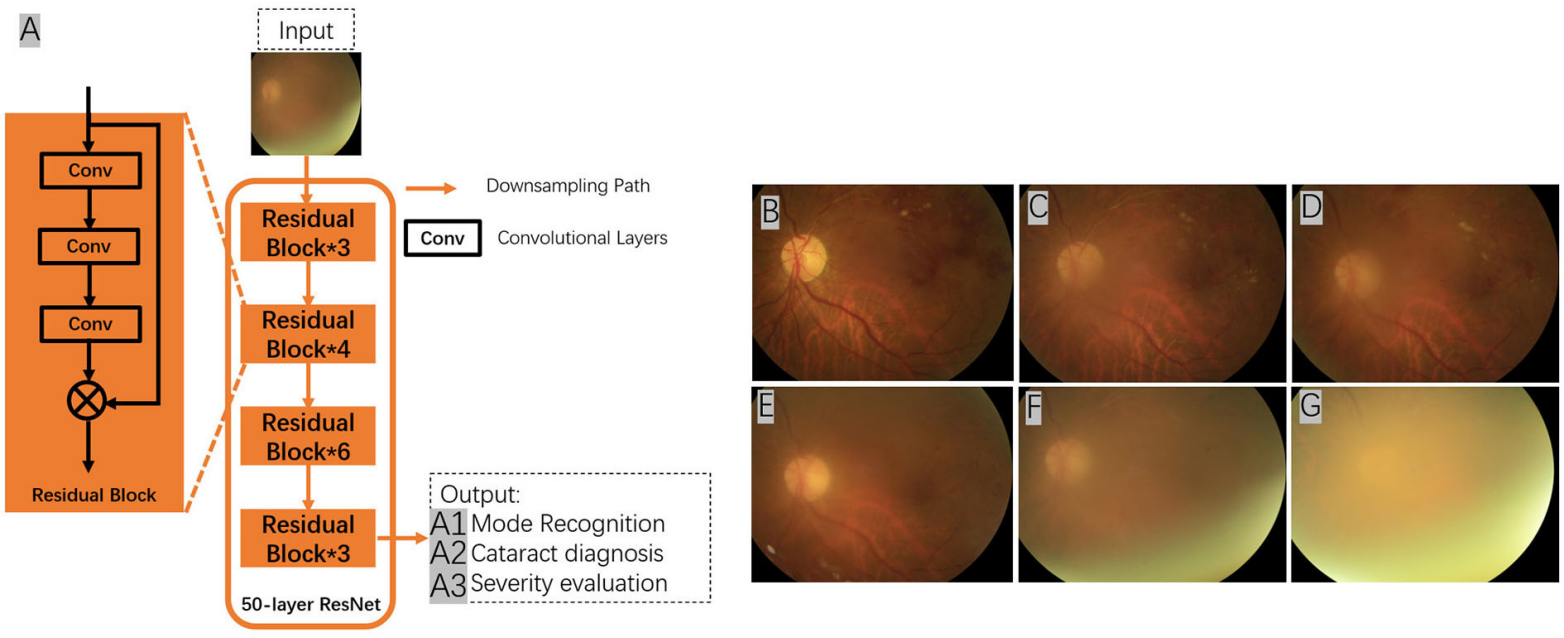
Using deep residual network (ResNet) for cataract recognition and grading. The overall architecture of the ResNet consists of 16 residual blocks and each residual block consists of three convolutional layers. The output of the ResNet includes: (A1) mode recognition - identify the capture mode between mydriatic and non-mydriatic images, and between optical section and diffuse slit lamp illimitation; (A2) cataract recognition - the system could classify the images as normal (no cataract), cataractous, or postoperative intraocular lens (IOL); and (A3) severity evaluation - classify the type and severity of the cataract **(A–G)**, and assess the subsequent follow-up or referral arrangements for the patient. Conv, convolutional layers.

AI algorithms are important for the automatic detection and grading of cataracts based on slit lamp photographs or color fundus photographs (Figure 2). Wu et al. (17) used a DL system for the diagnosis and referral of cataracts based on slit-lamp photographs. Three steps are performed sequentially in this system: (i) identify the capture mode between mydriatic and non-mydriatic images, and between optical section and diffuse slit lamp imaging; (ii) classify the images as normal (no cataract), cataractous, or postoperative intraocular lens (IOL); and (iii) classify the type and severity of the cataract or posterior capsular opacification and assess the subsequent follow-up or referral arrangements for the patient. The AUC of the CMAAI validation set was more than 99% for both capture mode recognition and cataract detection. For cataract severity evaluation, using mydriatic images with optical sections achieved the best performance (AUC 0.99), whereas using nonmydriatic images with diffuse illumination was less effective (AUC 0.9328).

A limited number of studies have been conducted on automated cataract assessment systems using color fundus photographs. Dong et al. (47) used a CNN for feature extraction and a SoftMax function for cataract detection and severity grading. Ran et al. (48) used a CNN and random forest for the same task. Pratap and Kokil (49) performed transfer learning, in which a pre-trained CNN was trained on natural images (non-medical images), which were further refined with 400 fundus images. The training and test data used in this study are available from open-source databases, including the high-resolution fundus image database (HRF), STARE, standard DR database calibration level 0 (DIARETDB0), methods to evaluate segmentation and indexing techniques in the field of retinal ophthalmology (MESSIDOR), DRIVE, fundus image registration dataset (FIRE), digital retinal images for optic nerve segmentation database (DRIONS_DB), and Indian diabetic retinopathy image dataset (IDRiD). Li et al. (50) developed a DL system using training data from the clinical database of the Beijing Tongren Eye Center. ResNet-18 and ResNet-50 were used for cataract detection and severity grading (non-cataracts, mild, moderate, and severe cataracts). Therefore, explainable attention maps can be used to illustrate the presence and severity of cataract.

The treatment strategy for cataracts is surgical removal accompanied by intraocular lens (IOL) implantation. AI has also been used to calculate IOL power, which significantly improves the prognosis and visual outcome of cataract surgery.

### Glaucoma

Glaucoma is an optic nerve degenerative disease characterized by typical pathological changes in the optic nerve head, retinal nerve fiber layer (RNFL), and visual field. Glaucoma is the second leading cause of irreversible blindness worldwide, and approximately 50% of glaucoma cases remain undiagnosed. Early diagnosis and intervention are essential for preventing blindness. Glaucoma can be classified as open-angle glaucoma or closure-angle glaucoma. Early diagnosis of glaucoma requires a combination of several examination results, including IOP, disc compression/decompression (C/D) ratio, morphology, visual field, and RNFL changes. The C/D ratio is a common index used to evaluate glaucomatous optic nerve damage. The difficulty of the computerized automatic diagnosis system is in segmenting the optic disc and optic cup areas from the fundus image. There is also an association between diabetes and the development of glaucoma, and screening for open-angle and closed-angle glaucoma in the population with diabetes is clinically and scientifically relevant.

The prerequisite for segmentation is localization. Researchers have recently proposed a method for localizing the optic disc based on vessel tracking, using a minimum variance classifier based to predict the region containing the optic disc. The connected partial markers and luminance information are used to identify the fundus vessels, which eventually assist to predict the optic disc (51). Other researchers created a comprehensive dataset of retinal images containing both normal and glaucomatous eyes, which were manually segmented by several ophthalmologists to provide information on other optic nerve head (ONH) regions, including disc rim cuts (52). This dataset is openly accessible and is anticipated to facilitate further research on glaucoma AI diagnosis.

Different imaging characteristics were thoroughly evaluated to determine the most significant characteristics of glaucoma. The researchers trained a multimodal model incorporating multiple deep neural networks and used it for the early detection of glaucoma by training macular volumes on OCT and color fundus photographs and combining demographic and clinical data. The accurate prediction of posttraumatic growth (PTG) through interpretable analysis highlighted the variables that change with the progression of glaucoma, including age and lung function (53). Other investigators have demonstrated the importance of the spatial structure of the thickness map data of the retinal neural fiber layer in the diagnosis of glaucoma using multiple ML models, including two traditional ML algorithms, the support vector machine (SVM) and K-nearest neighbor (KNN), as well as two CNNs, ResNet-18 and Glaucoma Net, to detect glaucoma diagnostic accuracy and support further efforts to optimize the use of these data (54).

Christopher (55) evaluated the ability of DL methods to identify glaucomatous optic neuropathy (GON) using fundus photographs. Two independent ophthalmologists evaluated a large database of fundus photographs of a racially and ethnically diverse group of individuals. The best DL model achieved an AUC of 0.91 in distinguishing GON eyes from healthy eyes, 0.97 for identifying GON eyes with moderate-to-severe functional loss, and 0.89 for GON eyes with mild functional loss. The visualization results indicated that the DL model focused on the anatomical features of the inferior and superior regions of the optic disc. These results suggest that the DL-based assessment of fundus images could be useful in the automation of large-scale glaucoma detection and screening programs. Shibata et al. (56) also developed a deep residual learning algorithm to screen for glaucoma using fundus photography and measured its diagnostic performance compared with that of ophthalmology residents. The DL algorithm achieved a significantly higher diagnostic performance than residents in ophthalmology. Berchuck et al. (57) developed a DL algorithm to improve the estimation of the rate of progression of glaucoma vision loss and the prediction of future patterns. A low-dimensional representation of the standard automatic visual field (SAP) was learned by training a generalized variational self-encoder (VAE) using 29161 visual fields from 3,832 patients. The VAE was trained with 90% of the data sample and randomized at the patient level. Using the remaining 10%, progression rates and predictions were generated and compared to SAP mean deviation (MD) rates and point-by-point (PW) regression predictions, respectively. Longitudinal rates of change through the VAE latent space detected significantly higher rates of progression than MD at 2 and 4 years after baseline. Deep VAE can be used to assess the incidence and trajectory of glaucoma and has an added benefit as a generative technique that can predict future patterns of visual field damage. Wu et al. (58) evaluated the effect of five glaucoma treatments (medication, laser, non-laser surgery (NLS), laser + medication, and NLS + medication) on a 1-year IOP change, which provides important evidence of clinical outcomes for glaucoma patients. Li et al. (59) developed and evaluated the performance of “iGlaucoma,” a smartphone application-based DL system in detecting visual field (VF) changes in glaucoma. In this study, which was divided into two phases, 1,614,808 data points from 10,784 VF (5 542 patients) from seven centers in China were included. The first phase involves training, validating, and testing the diagnostic performance of the DL system. In the second phase, the iGlaucoma cloud-based application was further tested with 33,748 data points from 649 VFs from 437 patients from three glaucoma clinics. In the second stage, the accuracy of iGlaucoma for identifying different patterns in the probability plot region of pattern deviation was 0.99, and the corresponding AUC, sensitivity, and specificity were 0.966 (0.953–0.979), 0.954 (0.930–0.977), and 0.873 (0.838–0.908), respectively.

A longitudinal dataset combining VF and clinical data was used to evaluate the performance of the convolutional long short-term memory (LSTM) neural network. Models trained on VF and clinical data (AUC, 0.89–0.93) performed better than models trained on VF results only (AUC, 0.79–0.82; *P* < 0.001), demonstrating that supplementing VF results with clinical data improves the ability of the model to assess glaucoma progression (60). Furthermore, the investigator validated the traditional artificial neural networks and discovered that they can perform well in detecting spinal field defects in glaucoma cohorts and in detecting visual field defects caused by pituitary disease in a glaucoma population (60). Other researchers have developed hybrid deep learning model (HDLM) algorithms that can quantitatively predict the thickness of the macular ganglion intracellular reticular layer (mGCIPL) from non-red retinal neurofibrillary layer photographs (RNFLPs) with good performance (61). Researchers developed a DL algorithm called image ResNet to discriminate glaucoma and obtained test data with an area under the curve (ROC) of 96.5 (95% confidence interval [CI]: 93.5–99.6), indicating that the DL algorithm outperformed ophthalmology residents in diagnosis (56). The investigators evaluated the external validity of the dynamic structure–function (DSF) model through studies tested in an independent dataset (intraocular pressure treatment study-focal scanning laser fundoscopy [OHTS-CSLO]-assisted study; *N* = 178 eyes) and the Glaucoma Diagnostic Innovations Study or the African Descent and Glaucoma Assessment Study (DIGS/ADAGES) dataset, demonstrating the external validity of the DSF model and its potential to develop it into a useful clinical tool (62). Some investigators have demonstrated the value of ML models in predicting trabeculectomy outcomes in patients with refractory glaucoma using models of random forests, SVMs, artificial neural networks, and multivariate logistic regression to predict the surgical outcome of trabeculectomy (63). A Bayesian deep multi-source learning (BDMSL) model is proposed, which introduces an information-centric multi-source learning framework to integrate multi-source data while employing Bayesian DL to obtain uncertainty information of the model and achieve better performance than other methods (64). The CNN was trained using OCT images and adjusted by the Humphrey field analyzer (HFA) 24–2 to establish a prediction model of the 10-degree central field of VF for glaucoma patients (65). The researchers have also used the DL model that uses fundus photographs to detect superficial anterior chamber depth (ACD) as a screening tool for angle-closure glaucoma (ACG). The cycle generative adversarial network (cycle GAN)—based feature maps show hidden features of superficial ACD that are undetectable by traditional techniques and ophthalmologists and help detect early ACD (66). Some investigators have analyzed multiple features and introduced new cross-sectional ONH features from OCT images to facilitate the current diagnostic evaluation of glaucoma, demonstrating that selected features and cross-sectional ONH cup areas trained using DL have great potential as preliminary screening tools for glaucoma (67). These results will help clinicians make more accurate decisions in the future.

The investigators developed and evaluated the performance of a DL system based on a smartphone app through efficient glaucoma diagnostic workers based on VFs, providing keening to detect visual field changes in glaucoma with smartphones (67). Glaucoma is a disease associated with the loss of retinal ganglion cells (RGCs). The main research efforts are currently being conducted with the help of rodent models, making a tool that reliably quantifies the survival of RGCs. Therefore, some researchers have designed software called RGCode (DL-based quantification of RGCs), which is capable of fully automated RGC quantification in the entire mouse retina (68). Researchers have developed a non-species specificity, which can be extended to the tools of glaucoma AxoNet. It can be calculated from various animal models of glaucoma RGC axons in the optic nerve (ON) organization image, and use the depth study to return to the pixel-level counting axon density estimation and then integrate it into the image area to determine the axon count (68).

### Age-related macular degeneration

**Figure 3:**
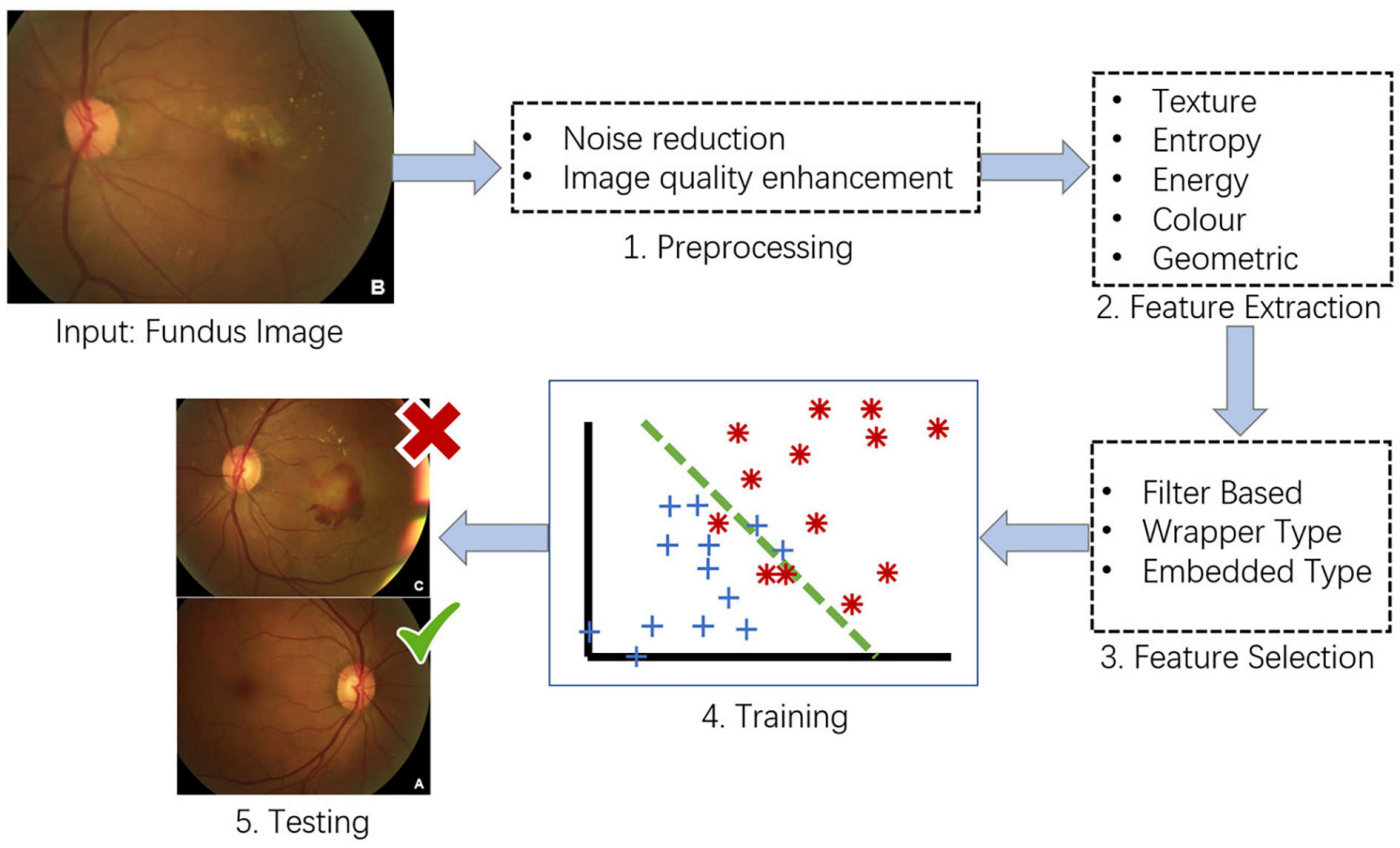
Conventional framework for ARMD detection from fundus images. **(1)** Preprocessing – image preprocessing is performed on the input fundus image to reduce noise and enhance image quality. **(2)** Feature Extraction – image features such as texture, entropy and color features will be extracted from the preprocessed images. **(3)** Feature Selection – feature selection will be conducted on the extracted image features to select the best representative features of an image. **(4)** Training – at the training phase, a model such as support vector machine (SVM) will be built that tries to separate the training data into different categories e.g., ARMD and non-ARMD. **(5)** Testing – testing phase will apply the trained model to unseen fundus images and classify them to the known categories e.g., ARMD and non-ARMD.

ARMD is an acquired and complex macular degenerative disease that is the leading cause of blindness in the elderly worldwide. The prevalence increases exponentially every decade after the age of 50 (69). Aging, smoking, genetic susceptibility, dysregulated lipid metabolism, oxidative stress, cardiovascular disease, female sex, white race, obesity hyperopia, and other risk factors contribute to ARMD development. The clinical characteristics include the presence of drusen, retinal pigment epithelium (RPE) abnormalities, geographical atrophy, and neovascular derangement. ARMD can be classified into early ARMD [characterized by numerous small (< 63 microns, hard) or intermediate (≥63 microns but <125 microns, soft) drusen]; intermediate ARMD [defined by extensive drusen with small or intermediate size, or any large drusen (≥125 microns)]; and advanced ARMD (characterized by a choroidal neovascular membrane or geographic atrophy). ARMD can be categorized into two subtypes: dry (presence of drusen, RPE abnormalities, or geographical atrophy) or wet (macular neovascularization). The diagnosis of ARMD frequently relies on various examinations, such as fundus photography, fundus fluorescein angiography, indocyanine green angiography, OCT, and OCTA. Early- and mid-stage ARMD can be asymptomatic, leading to easy underdiagnosis, while advanced ARMD progresses faster and has a greater impact on vision, with limited treatment options available (Figure 3). AI can be an essential tool for the early identification of macular lesions that can assist ophthalmologists in the early intervention of the disease.

Recent studies have proposed DL algorithms based on fundus color photography to identify drusen or retinal pigment epithelium (RPE) abnormalities in ARMD. Researchers from Johns Hopkins University achieved an accuracy of 88.1–91.6% for the identification of drusen, which is competitive with manual interpretation (13, 15, 16). AI based on convolutional neural networks (CNNs) has also been used for telemedicine. In this study, an annotated dataset consists of 35,900 ARMD OCT images (acquired from two types of OCT devices including Zeiss Cirrus HD-OCT 4000 and Optovue RTVue-XR Avanti) was used for AI algorithm training and validation groups, respectively, and the CNN architectures named ResNet 50, Inception V3, and VGG 16 were used for image recognition. The detection accuracy of the AI-based system achieved the same image discrimination rate as that of retinal specialists (92.73 vs. 91.9%, *p* = 0.99) and generally higher than that of medical students (69.4 and 68.9%) (70). However, the testing performance of current AI algorithms is still largely dependent on different clinical datasets; therefore, their generalization performance among external clinical datasets is limited. Future work on the applicability and portability of these algorithms remains challenging.

**Figure 4:**
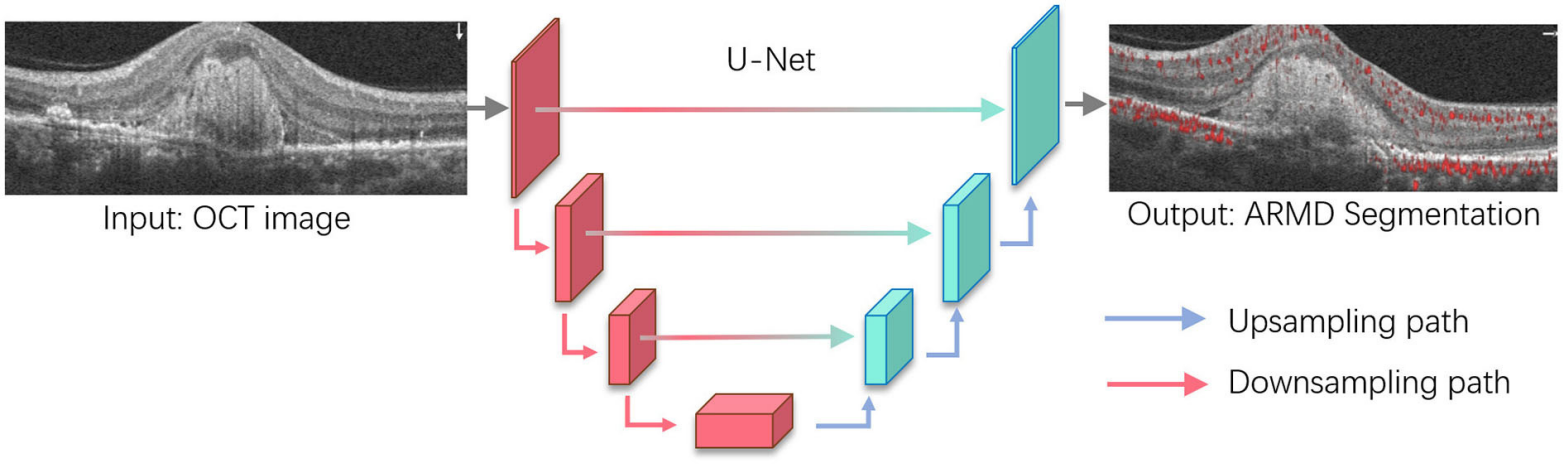
Age-related macular degeneration (ARMD) lesions segmentation based on U-Net. U-Net is one of the most widely used segmentation architectures for biomedical images and stemmed from the fully convolutional network. The U-Net model consists of a downsampling path and upsampling path, where downsampling path has convolutional and max-pooling layers to extract high-level abstract information while the upsampling path has convolutional and deconvolutional layers that upsample the feature maps to output the segmentation outcomes. For ARMD segmentation, U-Net will take OCT images as the input and progressively extract semantic features that allow to separate the lesions from the surrounding background and output the lesion segmentation results.

Owing to the high reliance on OCT images for the diagnosis of the wet form of ARMD (Figure 4), the recognition of ML is no longer limited to color fundus photography. AI research is beginning to focus on large databases of multimodal images and is expected to uncover more adequate information. Several intelligent decision systems based on OCT technology have been developed using ML (41). Meanwhile, the DL technique has achieved higher accuracy in distinguishing a healthy fundus from exudative ARMD (71). Related AI research teams have developed algorithms for the simultaneous recognition of multiple disease types, including macular edema, ARMD, and central serous choroidal retinopathy, which can not only discriminate the presence of retinopathy in the subject but also further indicate the type of retinopathy with satisfactory accuracy (72). This suggests that OCT is a natural fit for AI in the detection of macular diseases. Progression to exudative “wet” age-related macular degeneration (wARMD) is a major cause of visual impairment. For patients with unilateral eye wARMD, Yim et al. (73) introduced an AI system to predict the progression to wARMD of another eye using OCT images and corresponding automatic tissue maps. This system predicts conversion to exARMD within a clinically actionable 6-month time window and demonstrates the potential of using AI to predict disease progression.

Other studies have combined multimodal data to predict ARMD progression. Banerjee et al. (74) proposed a hybrid sequential prediction model called “Deep Sequence” that integrates radionics-based imaging features, demographic, and visual factors, with a recurrent neural network (RNN) model to predict the risk of exudation within a future time frame in non-exudative ARMD eyes. The proposed model provides scores associated with the risk of exudation in the short term (within 3 months) and long term (within 21 months), which allows for addressing challenges related to the variability of OCT scan characteristics and the size of the training cohort. Thakoor et al. (75) proposed a DL approach for multi-class detection of non-ARMD vs. non-neovascular (NNV) ARMD vs. NV ARMD from a combination of OCTA, OCT structure, 2D B-scan flow images, and high-definition (HD) 5-line b-scan cubes. DL also detects ocular biomarkers indicative of ARMD risk. Choroidal neovascularization and geographic atrophy were found to be significant biomarkers for ARMD detection by both CNNs and clinical experts. Detection of ARMD and its biomarkers from OCTA images *via* CNNs has tremendous potential to expedite the screening of patients with early and late-stage ARMD. Yeh et al. (76) proposed a heterogeneous data fusion network (HDF-Net) to predict visual acuity (VA) and to evaluate the prognosis and risk of progression of neovascular age-related macular degeneration (nARMD). The clinical decision-making process was simulated using a mixture of pre-processed information from raw OCT images and digital data, and HDF-Net performed well in predicting individualized treatment outcomes. This new approach is an important step toward personalized treatment strategies for typical nARMD.

Genetic and environmental factors influence the etiology of ARMD. Genome-wide association studies (GWAS) for late-stage ARMD have identified 52 independent genetic variants with genome-wide significance at 34 genomic loci. Yan et al. (77) used the Age-Related Eye Disease Study (AREDS) dataset and a modified CNN with genotype and fundus images to predict whether an eye had progressed to advanced ARMD, showing that the CNN with fundus images plus genotype achieved a mean AUC of 0.85 in predicting the progression of advanced ARMD, while the CNN with fundus images only achieved a mean AUC of 0.81. Strunz et al. (78) conducted a transcriptome-wide association study (TWAS) that predicted the impact of ARMD-associated genetic variants on gene expression, which addressed the shortcomings of current GWAS analyses that rarely identify functional variants associated with specific genes in the disease process. This study further highlights the fact that the expression of genes associated with ARMD is not restricted to retinal issues but is a systemic pathology.

### Other ocular diseases

In addition to the common ocular diseases discussed above, AI has shown promise in the diagnosis of the epidermal membrane (ERM), chronic central serous chorioretinopathy (CSC), bacterial keratitis (BK), pathological myopia, and macular edema (ME). Furthermore, ophthalmic image for AI analysis is not limited to color fundus photography but covers various ophthalmic images, including anterior segment photography, corneal topography, anterior or posterior segment OCT, and ultrasound biomicroscopy (UBM) (79).

A deep neural network-based AI model has been applied for epidermal membrane (ERM) detection based on color fundus photographs (80, 81). A random forest-based regression model was used to infer local retinal sensitivity from the retinal structure and the model was applied to the CSC patients for personalized treatment (81). Yoon et al. (82) used convolutional neural networks and achieved performance of 93.8, 90.0, 99.1, and 98.9% in accuracy, sensitivity, specificity, and AUC for the diagnosis of CSC. Kuo et al. (83) evaluated various DL algorithms, including ResNet50, ResNeXt50, DenseNet121, SE-ResNet50, EfficientNets, and DeepLab framework, and identified that DL algorithms could accurately diagnose BK based on eye anterior segment photographs (84). Besides, the DL algorithm has also been applied to ultra-wide-field fundus (UWF) images for the detection of ME and retinal exudates (85).

### Challenges of artificial intelligence in the medical field

Although the application of AI technology in the medical field, particularly in ophthalmology, is becoming more widespread, many problems need to be solved with the application of AI technology in current clinical practice. OCT is an indispensable component of healthcare in ophthalmology and plays a significant role in the diagnosis, grading, and assessment of treatment responses in eye diseases. These challenges can be attributed to the fact that eye diseases have various imaging characteristics, such as size and shape, fuzzy boundaries, low contrast to the surrounding background, and heterogeneity. These challenges have motivated the development of numerous AI-aided systems that can assist clinicians in image interpretation and offer opportunities to enhance clinical analytics and decision-making.

### Data quality control

Because the use of AI technology is predicated on a large amount of treatment data, the corresponding labels and data quality directly determine the performance of the model to an extent. Data quality may have the following problems: (i) poor quality of the data itself, such as blurred pictures and artifacts; (ii) poor quality of the data labels, such as incorrect labels; and (iii) insufficient data, where only a small portion of data has been labeled.

### Privacy protection

Cloud-based data management and storage platforms are commonly used to facilitate data acquisition across multiple cohorts, such as multiple hospitals. Data security in AI algorithms presents a significant challenge.

### Establishment of laws and regulations

The application of AI in ocular diseases remains a big challenge. Erroneous predictions by AI algorithms e.g., due to poor data quality, are unavoidable, which can lead to liability issues for physicians. Therefore, the role of physicians in the perspective of AI diagnosis and treatment process needs to be further refined in future medical regulations. In addition, the compliance of different AI algorithms for the diagnosis of various ocular diseases would also require dedicated regulations. In July 2019, to strengthen the guidance of the registration declaration of AI medical devices and further improve the quality of the review, the State Drug Administration Medical Device Technical Review Center in China organized the development of “DL-assisted decision-making medical device software review points.” On January 15, 2020, the State Drug Administration reviewed and approved the first artificial intelligence Class III medical device ”Coronary Blood Flow Reserve Fraction Calculation Software“ in China. The product is based on coronary CT vessel images and consists of an installation CD and encryption lock. The functional modules include basic image operations, vessel segmentation, and reconstruction based on DL technology, vessel centerline extraction, and blood flow reserve fraction calculation based on DL technology, which pioneered the application of domestic artificial intelligence-aided diagnosis and treatment software in clinical practice. Internationally, the U.S. FDA approved IDx's Idx-DR DR screening software in April 2018, which detects the severity of glucose retinal symptoms in adult patients with diabetes based on fundus photographs, and provides recommendations on whether a referral for examination is needed. This is the first product approved by the U.S. FDA using a new generation of AI technology for glucose retinal screening software, and the review and approval of its products will help further promote the approval and supervision of AI-aided diagnostic software for diabetic fundus disease in China. Existing silicon-based intelligence, somatotropic technology, Shanggong Medical Information, Deep et al. (39), and many other diabetes AI-aided diagnostic products have been actively involved in registration declarations. The means and efficiency of DR screening and auxiliary diagnosis are expected to become more efficient and accurate in the future.

### Lack of clinical context

AI programs are driven by data interpretation, and frequently lack consideration of the underlying clinical context. In particular, AI programs have difficulty holistically processing clinical scenarios, nor can they fully account for the psychological and social aspects of human nature that skilled physicians would normally consider (86). Cabitza et al. discussed the importance of clinical settings and provided an example of an ML prognostic model that, although technically valid, led to the interpretation of clinical data for treating patients with pneumonia. The AI program, which targeted 14 199 patients with pneumonia, showed that those with asthma had a lower risk of dying of pneumonia than those without concurrent asthma. The correctly coded program predicted asthma as a protective feature because asthma patients are frequently admitted to the intensive care unit (ICU) to prevent complications; however, mortality in ICU patients was 50% lower than in patients with pneumonia alone, and patients with asthma and pneumonia had a better prognosis than those with pneumonia alone (86, 87).

## Future directions

AI technology has made significant progress not only in treating ophthalmic diseases but also in other systemic diseases with initial results. The direct observation of retinal vessels in the fundus, combined with several physiological and biochemical indicators of the entire body supplemented with AI algorithms for learning and analysis, provides a new method for evaluating risk factors for cardiovascular diseases. In the management of patients with diabetes, it can also be used to predict the risk factors for diabetes-related complications (diabetic nephropathy, cardiovascular disease, diabetic peripheral neuropathy, etc.). Establishing a model to identify complicated eye diseases (DR complicated with glaucoma or cataract) with multiple imaging modalities, such as OCT, OCTA, and fundus photography, is highly desirable. Although there are still some challenges in current clinical practice, the promising developments demonstrated with AI technology in the above applications suggest that it will be of great clinical significance in the future.

## Author contributions

XZ and BS: conceptualization. BS, XC, TL, TM, LB, YY, and XZ: writing. XZ, LB, and BS: review and editing. All authors contributed to the article and approved the submitted version.

## Funding

This work was supported by the National Natural Science Foundation of China (Grant 81570850, 82070988, and 62272298), the Ministry of Science and Technology Foundation of China (Grants 2016YFC1305604), the Shanghai Municipal Science and Technology Major Project under Grant (Grant 2021SHZDZX0102), the College-level Project Fund of Shanghai Jiao tong University Affiliated Sixth People's Hospital (ynlc201909), and the Medical-industrial Cross-fund of Shanghai Jiao Tong University (YG2022QN089).

## Conflict of interest

The authors declare that the research was conducted in the absence of any commercial or financial relationships that could be construed as a potential conflict of interest.

## Publisher's note

All claims expressed in this article are solely those of the authors and do not necessarily represent those of their affiliated organizations, or those of the publisher, the editors and the reviewers. Any product that may be evaluated in this article, or claim that may be made by its manufacturer, is not guaranteed or endorsed by the publisher.

## References

[1] McCarthy J Minsky ML Rochester N Shannon CE. A proposal for the dartmouth summer research project on artificial intelligence. AI Mag. (2006) 27:12. 10.1007/978-1-4613-8716-9

[2] Samuel AL. Some studies in machine learning using the game of checkers. II—recent progress. Comput Games I. (1988) 1:366–400. 10.1007/978-1-4613-8716-9_15

[3] LeCun Y Bengio Y Hinton G. Deep learning. Nature. (2015) 521:436–44. 10.1038/nature14539 26017442

[4] Lee CS Tyring AJ Deruyter NP Wu Y Rokem A Lee AY. Deep-learning based, automated segmentation of macular edema in optical coherence tomography. Biomed Opt Express. (2017) 8:3440–8. 10.1364/BOE.8.003440 28717579 PMC5508840

[5] Born J Beymer D Rajan D Coy A Mukherjee VV Manica M. On the role of artificial intelligence in medical imaging of COVID-19. Patterns. (2021) 2:100269. 10.1016/j.patter.2021.100269 33969323 PMC8086827

[6] Chi J Walia E Babyn P Wang J Groot G Eramian M. Thyroid nodule classification in ultrasound images by fine-tuning deep convolutional neural network. J Digit Imaging. (2017) 30:477–86. 10.1007/s10278-017-9997-y 28695342 PMC5537102

[7] Song J Chai YJ Masuoka H Park SW Kim SJ Choi JY. Ultrasound image analysis using deep learning algorithm for the diagnosis of thyroid nodules. Medicine. (2019) 98:e15133. 10.1097/MD.0000000000015133 30985680 PMC6485748

[8] Setio AA Ciompi F Litjens G Gerke P Jacobs C van Riel SJ. Pulmonary nodule detection in CT images: false positive reduction using multi-view convolutional networks. IEEE Trans Med Imaging. (2016) 35:1160–9. 10.1109/TMI.2016.2536809 26955024

[9] Ding J Li A Hu L Z Wang. Accurate pulmonary nodule detection in computed tomography images using deep convolutional neural networks. In:Descoteaux M Maier-Hein L Franz A Jannin P Collins D Duchesne S, editors. Lecture Notes in Computer Science. Cham: Springer. (2017). p. 559–67.

[10] Abràmoff MD Lou Y Erginay A Clarida W Amelon R Folk JC. Improved automated detection of diabetic retinopathy on a publicly available dataset through integration of deep learning. Invest Ophthalmol Vis Sci. (2016) 57:5200–6. 10.1167/iovs.16-19964 27701631

[11] Gargeya R Leng T. Automated identification of diabetic retinopathy using deep learning. Ophthalmology. (2017) 124:962–9. 10.1016/j.ophtha.2017.02.008 28359545

[12] Gulshan V Peng L Coram M Stumpe MC Wu D Narayanaswamy A. Development and validation of a deep learning algorithm for detection of diabetic retinopathy in retinal fundus photographs. JAMA. (2016) 316:2402–10. 10.1001/jama.2016.17216 27898976

[13] Ting DSW Cheung CYL Lim G Tan GSW Quang ND Gan A. Development and validation of a deep learning system for diabetic retinopathy and related eye diseases using retinal images from multiethnic populations with diabetes. JAMA. (2017) 318:2211–23. 10.1001/jama.2017.18152 29234807 PMC5820739

[14] Li F Wang Z Qu G Song D Yuan Y Xu Y. Automatic differentiation of Glaucoma visual field from non-glaucoma visual filed using deep convolutional neural network. BMC Med Imaging. (2018) 18:35. 10.1186/s12880-018-0273-5 30286740 PMC6172715

[15] Burlina PM Joshi N Pekala M Pacheco KD Freund DE Bressler NM. Automated grading of age-related macular degeneration from color fundus images using deep convolutional neural networks. JAMA Ophthalmol. (2017) 135:1170–6. 10.1001/jamaophthalmol.2017.3782 28973096 PMC5710387

[16] Grassmann F Mengelkamp J Brandl C Harsch S Zimmermann ME Linkohr B. A deep learning algorithm for prediction of age-related eye disease study severity scale for age-related macular degeneration from color fundus photography. Ophthalmology. (2018) 125:1410–20. 10.1016/j.ophtha.2018.02.037 29653860

[17] Wu X Huang Y Liu Z Lai W Long E Zhang K. Universal artificial intelligence platform for collaborative management of cataracts. Br J Ophthalmol. (2019) 103:1553–60. 10.1136/bjophthalmol-2019-314729 31481392 PMC6855787

[18] Varadarajan AV Poplin R Blumer K Angermueller C Ledsam J Chopra R. Deep learning for predicting refractive error from retinal fundus images. Invest Ophthalmol Vis Sci. (2018) 59:2861–8. 10.1167/iovs.18-23887 30025129

[19] Brown JM Campbell JP Beers A Chang K Ostmo S Chan RVP. Automated diagnosis of plus disease in retinopathy of prematurity using deep convolutional neural networks. JAMA Ophthalmol. (2018) 136:803–10. 10.1001/jamaophthalmol.2018.1934 29801159 PMC6136045

[20] Ohsugi H Tabuchi H Enno H Ishitobi N. Accuracy of deep learning, a machine-learning technology, using ultra-wide-field fundus ophthalmoscopy for detecting rhegmatogenous retinal detachment. Sci Rep. (2017) 7:9425. 10.1038/s41598-017-09891-x 28842613 PMC5573327

[21] Masood S Fang R Li P Li H Sheng B Mathavan A. Automatic choroid layer segmentation from optical coherence tomography images using deep learning. Sci Rep. (2019) 9:3058. 10.1038/s41598-019-39795-x 30816296 PMC6395677

[22] Liu TYA Correa ZM. Deep learning applications in ocular oncology. In:Grzybowski A, editor. Artificial intelligence in ophthalmology. Cham: Springer. (2021). p. 235–8.

[23] Liu R Wang X Wu Q Dai L Fang X Yan T. DeepDRiD: Diabetic retinopathy—grading and image quality estimation challenge. Patterns. (2022) 3:100512. 10.1016/j.patter.2022.100512 35755875 PMC9214346

[24] Association AD. Microvascular complications and foot care: standards of medical care in diabetes-2021. Diabetes Care. (2021) 44:151–67. 10.2337/dc21-S011 33298422

[25] van der Heijden AA Abramoff MD Verbraak F van Hecke MV Liem A Nijpels G. Validation of automated screening for referable diabetic retinopathy with the IDx-DR device in the Hoorn Diabetes Care System. Acta Ophthalmol. (2018) 96:63–8. 10.1111/aos.13613 29178249 PMC5814834

[26] Yin B Li H Sheng B Hou X Chen Y Wu W. Vessel extraction from non-fluorescein fundus images using orientation-aware detector. Med Image Anal. (2015) 26:232–42. 10.1016/j.media.2015.09.002 26474120

[27] Reddy SS Sethi N Rajender R Mahesh G. Extensive analysis of machine learning algorithms to early detection of diabetic retinopathy. Mater Today. (2020). 10.1016/j.matpr.2020.10.894. Available online at: https://www.sciencedirect.com/science/article/pii/S221478532038531X (accessed December 26, 2020).

[28] Raman R Srinivasan S Virmani S Sivaprasad S Rao C Rajalakshmi R. Fundus photograph-based deep learning algorithms in detecting diabetic retinopathy. Eye (Lond). (2019) 33:97–109. 10.1038/s41433-018-0269-y 30401899 PMC6328553

[29] Babenko B Mitani A Traynis I Kitade N Singh P Maa AY. Detection of signs of disease in external photographs of the eyes via deep learning. Nat Biomed Eng. (2022) 1–14. 10.1038/s41551-022-00867-5. Available online at: https://www.nature.com/articles/s41551-022-00867-5#citeas (accessed March 29 2022). PMC8963675 35352000

[30] Keel S Lee PY Scheetz J Li Z Kotowicz MA MacIsaac RJ. Feasibility and patient acceptability of a novel artificial intelligence-based screening model for diabetic retinopathy at endocrinology outpatient services: a pilot study. Sci Rep. (2018) 8:4330. 10.1038/s41598-018-22612-2 29531299 PMC5847544

[31] Kermany DS Goldbaum M Cai W Valentim CCS Liang H Baxter SL. Identifying medical diagnoses and treatable diseases by image-based deep learning. Cell. (2018) 172:1122–31.e9. 10.1016/j.cell.2018.02.010 29474911

[32] Holmberg OG Köhler ND Martins T Siedlecki J Herold T Keidel L. Self-supervised retinal thickness prediction enables deep learning from unlabelled data to boost classification of diabetic retinopathy. Nat Mach Intell. (2020) 2:719–26. 10.1038/s42256-020-00247-1

[33] Sharafeldeen A Elsharkawy M Khalifa F Soliman A Ghazal M Alhalabi M. Precise higher-order reflectivity and morphology models for early diagnosis of diabetic retinopathy using OCT images. Sci Rep. (2021) 11:4730. 10.1038/s41598-021-83735-7 33633139 PMC7907116

[34] Abràmoff MD Lavin PT Birch M Shah N Folk JC. Pivotal trial of an autonomous AI-based diagnostic system for detection of diabetic retinopathy in primary care offices. NPJ Digit Med. (2018) 1:39. 10.1038/s41746-018-0040-6 31304320 PMC6550188

[35] Sahlsten J Jaskari J Kivinen J Turunen L Jaanio E Hietala K. Deep learning fundus image analysis for diabetic retinopathy and macular edema grading. Sci Rep. (2019) 9:10750. 10.1038/s41598-019-47181-w 31341220 PMC6656880

[36] Li F Wang Y Xu T Dong L Yan L Jiang M. Deep learning-based automated detection for diabetic retinopathy and diabetic macular oedema in retinal fundus photographs. Eye (Lond). (2021) 36:1433–41. 10.1038/s41433-021-01552-8 34211137 PMC9232645

[37] Reguant R Brunak S Saha S. Understanding inherent image features in CNN-based assessment of diabetic retinopathy. Sci Rep. (2021) 11:9704. 10.1038/s41598-021-89225-0 33958686 PMC8102512

[38] Ryu G Lee K Park D Park SH Sagong M. A deep learning model for identifying diabetic retinopathy using optical coherence tomography angiography. Sci Rep. (2021) 11:23024. 10.1038/s41598-021-02479-6 34837030 PMC8626435

[39] Dai L Wu L Li H Cai C Wu Q Kong H. A deep learning system for detecting diabetic retinopathy across the disease spectrum. Nat Commun. (2021) 12:3242. 10.1038/s41467-021-23458-5 34050158 PMC8163820

[40] Yau JW Rogers SL Kawasaki R Lamoureux EL Kowalski JW Bek T. Global prevalence and major risk factors of diabetic retinopathy. Diabetes Care. (2012) 35:556–64. 10.2337/dc11-1909 22301125 PMC3322721

[41] ElTanboly A Ismail M Shalaby A Switala A El-Baz A Schaal S. A computer-aided diagnostic system for detecting diabetic retinopathy in optical coherence tomography images. Med Phys. (2017) 44:914–23. 10.1002/mp.12071 28035657

[42] Al-Mukhtar M Morad AH Albadri M Islam MDS. Weakly supervised sensitive heatmap framework to classify and localize diabetic retinopathy lesions. Sci Rep. (2021) 11:23631. 10.1038/s41598-021-02834-7 34880311 PMC8655092

[43] Rajalakshmi R Subashini R Anjana RM Mohan V. Automated diabetic retinopathy detection in smartphone-based fundus photography using artificial intelligence. Eye (Lond). (2018) 32:1138–44. 10.1038/s41433-018-0064-9 29520050 PMC5997766

[44] Kumari S Venkatesh P Tandon N Chawla R Takkar B Kumar A. Selfie fundus imaging for diabetic retinopathy screening. Eye (Lond). (2021) 36:1988–93. 10.2139/ssrn.3785992 34642496 PMC8505467

[45] Zhang K Liu X Xu J Yuan J Cai W Chen T. Deep-learning models for the detection and incidence prediction of chronic kidney disease and type 2 diabetes from retinal fundus images. Nat Biomed Eng. (2021) 5:533–45. 10.1038/s41551-021-00745-6 34131321

[46] Müller-Breitenkamp U Ohrloff C Hockwin O. Aspects of physiology, pathology and epidemiology of cataract. Ophthalmologe. (1992) 89:257–67. 1304195

[47] Dong Y Zhang Q Qiao Z Yang J. Classification of cataract fundus image based on deep learning. In: 2017 IEEE International Conference on Imaging Systems and Techniques (IST). Beijing, China: IEEE. (2017).

[48] Ran J Niu K He Z Zhang H Song H. Cataract detection and grading based on combination of deep convolutional neural network and random forests. In: 2018 International Conference on Network Infrastructure and Digital Content (IC-NIDC). Guiyang, China: IEEE. (2018).

[49] Pratap T Kokil P. Computer-aided diagnosis of cataract using deep transfer learning. Biomed Signal Process Control. (2019) 53:101533. 10.1016/j.bspc.2019.04.010

[50] Li J Xu X Guan Y Imran A Liu B Zhang L., editors. Automatic cataract diagnosis by image-based interpretability. In: 2018 IEEE International Conference on Systems, Man, and Cybernetics (SMC). (2018) Miyazaki, Japan: IEEE.

[51] Wang R Zheng L Xiong C Qiu C Li H Hou X. Retinal optic disc localization using convergence tracking of blood vessels. Multimed Tools Appl. (2017) 76:23309–31. 10.1007/s11042-016-4146-z

[52] Sivaswamy J Krishnadas SR Chakravarty A Joshi GD. A comprehensive retinal image dataset for the assessment of glaucoma from the optic nerve head analysis. JSM Biomed Imaging Data Pap. (2015) 2:1004.

[53] Mehta P Petersen CA Wen JC Banitt MR Chen PP Bojikian KD. Automated detection of glaucoma with interpretable machine learning using clinical data and multimodal retinal images. Am J Ophthalmol. (2021) 231:154–69. 10.1016/j.ajo.2021.04.021 33945818 PMC8560651

[54] Wang P Shen J Chang R Moloney M Torres M Burkemper B. Machine learning models for diagnosing glaucoma from retinal nerve fiber layer thickness maps. Ophthalmol Glaucoma. (2019) 2:422–8. 10.1016/j.ogla.2019.08.004 32672575 PMC7368087

[55] Christopher M Belghith A Bowd C Proudfoot JA Goldbaum MH Weinreb RN. Performance of deep learning architectures and transfer learning for detecting glaucomatous optic neuropathy in fundus photographs. Sci Rep. (2018) 8:1–13. 10.1038/s41598-018-35044-9 30420630 PMC6232132

[56] Shibata N Tanito M Mitsuhashi K Fujino Y Matsuura M Murata H. Development of a deep residual learning algorithm to screen for glaucoma from fundus photography. Sci Rep. (2018) 8:14665. 10.1038/s41598-018-33013-w 30279554 PMC6168579

[57] Berchuck SI Mukherjee S Medeiros FA. Estimating rates of progression and predicting future visual fields in glaucoma using a deep variational autoencoder. Sci Rep. (2019) 9:18113. 10.1038/s41598-019-54653-6 31792321 PMC6888896

[58] Wu M Liu M Schuman JS Wang Y Lucy KA Ishikawa H. Evaluating glaucoma treatment effect on intraocular pressure reduction using propensity score weighted regression. Sci Rep. (2019) 9:15496. 10.1038/s41598-019-52052-5 31664148 PMC6820863

[59] Li F Song D Chen H Xiong J Li X Zhong H. Development and clinical deployment of a smartphone-based visual field deep learning system for glaucoma detection. NPJ Digit Med. (2020) 3:123. 10.1038/s41746-020-00329-9 33043147 PMC7508974

[60] Dixit A Yohannan J Boland MV. Assessing glaucoma progression using machine learning trained on longitudinal visual field and clinical data. Ophthalmology. (2021) 128:1016–26. 10.1016/j.ophtha.2020.12.020 33359887 PMC8222148

[61] Lee J Kim YK Ha A Sun S Kim YW Kim JS. Macular ganglion cell-inner plexiform layer thickness prediction from red-free fundus photography using hybrid deep learning model. Sci Rep. (2020) 10:3280. 10.1038/s41598-020-60277-y 32094401 PMC7039950

[62] Abu SL KhalafAllah MT Racette L. Evaluation of the external validity of a joint structure-function model for monitoring glaucoma progression. Sci Rep. (2020) 10:19701. 10.1038/s41598-020-76834-4 33184431 PMC7665194

[63] Banna HU Zanabli A McMillan B Lehmann M Gupta S Gerbo M. Evaluation of machine learning algorithms for trabeculectomy outcome prediction in patients with glaucoma. Sci Rep. (2022) 12:2473. 10.1038/s41598-022-06438-7 35169235 PMC8847459

[64] Chai Y Bian Y Liu H Li J Xue J. Glaucoma diagnosis in the Chinese context: an uncertainty information-centric Bayesian deep learning model. Inf Process Manag. (2021) 58:102454. 10.1016/j.ipm.2020.102454

[65] Asano S Asaoka R Murata H Hashimoto Y Miki A Mori K. Predicting the central 10 degrees visual field in glaucoma by applying a deep learning algorithm to optical coherence tomography images. Sci Rep. (2021) 11:2214. 10.1038/s41598-020-79494-6 33500462 PMC7838164

[66] Yoo TK Ryu IH Kim JK Lee IS Kim HK. A deep learning approach for detection of shallow anterior chamber depth based on the hidden features of fundus photographs. Comput Methods Programs Biomed. (2022) 219:106735. 10.1016/j.cmpb.2022.106735 35305492

[67] Akter N Fletcher J Perry S Simunovic MP Briggs N Roy M. Glaucoma diagnosis using multi-feature analysis and a deep learning technique. Sci Rep. (2022) 12:8064. 10.1038/s41598-022-12147-y 35577876 PMC9110703

[68] Masin L Claes M Bergmans S Cools L Andries L Davis BM. A novel retinal ganglion cell quantification tool based on deep learning. Sci Rep. (2021) 11:702. 10.1038/s41598-020-80308-y 33436866 PMC7804414

[69] Cheung LK Eaton A. Age-related macular degeneration. Pharmacotherapy. (2013) 33:838–55. 10.1002/phar.1264 23580402

[70] Hwang DK Hsu CC Chang KJ Chao D Sun CH Jheng YC. Artificial intelligence-based decision-making for age-related macular degeneration. Theranostics. (2019) 9:232–45. 10.7150/thno.28447 30662564 PMC6332801

[71] Treder M Lauermann JL Eter N. Automated detection of exudative age-related macular degeneration in spectral domain optical coherence tomography using deep learning. Graefes Arch Clin Exp Ophthalmol. (2018) 256:259–65. 10.1007/s00417-017-3850-3 29159541

[72] Khalid S Akram MU Hassan T Nasim A Jameel A. Fully automated robust system to detect retinal edema, central serous chorioretinopathy, and age related macular degeneration from optical coherence tomography images. Biomed Res Int. (2017) 2017:7148245. 10.1155/2017/7148245 28424788 PMC5382397

[73] Yim J Chopra R Spitz T Winkens J Obika A Kelly C. Predicting conversion to wet age-related macular degeneration using deep learning. Nat Med. (2020) 26:892–9. 10.1038/s41591-020-0867-7 32424211

[74] Banerjee I de Sisternes L Hallak JA Leng T Osborne A Rosenfeld PJ. Prediction of age-related macular degeneration disease using a sequential deep learning approach on longitudinal SD-OCT imaging biomarkers. Sci Rep. (2020) 10:15434. 10.1038/s41598-020-72359-y 32963300 PMC7508843

[75] Thakoor KA Yao J Bordbar D Moussa O Lin W Sajda P. A multimodal deep learning system to distinguish late stages of AMD and to compare expert vs. AI ocular biomarkers. Sci Rep. (2022) 12:2585. 10.1038/s41598-022-06273-w 35173191 PMC8850456

[76] Yeh T Luo A Deng Y Lee Y Chen S Chang P. Prediction of treatment outcome in neovascular age-related macular degeneration using a novel convolutional neural network. Sci Rep. (2022) 12:5871. 10.1038/s41598-022-09642-7 35393449 PMC8989893

[77] Yan Q Weeks DE Xin H Swaroop A Chew EY Huang H. Deep-learning-based prediction of late age-related macular degeneration progression. Nat Mach Intell. (2020) 2:141–50. 10.1038/s42256-020-0154-9 32285025 PMC7153739

[78] Strunz T Lauwen S Kiel C Hollander AD Weber BHF. A transcriptome-wide association study based on 27 tissues identifies 106 genes potentially relevant for disease pathology in age-related macular degeneration. Sci Rep. (2020) 10:1584. 10.1038/s41598-020-58510-9 32005911 PMC6994629

[79] Xiao S Bucher F Wu Y Rokem A Lee CS Marra KV. Fully automated, deep learning segmentation of oxygen-induced retinopathy images. JCI insight. (2017) 2:24. 10.1172/jci.insight.97585 29263301 PMC5752269

[80] Shao E Liu C Wang L Song D Guo L Yao X. Artificial intelligence-based detection of epimacular membrane from color fundus photographs. Sci Rep. (2021) 11:19291. 10.1038/s41598-021-98510-x 34588493 PMC8481557

[81] Pfau M van Dijk EHC van Rijssen TJ Schmitz-Valckenberg S Holz FG Fleckenstein M. Estimation of current and post-treatment retinal function in chronic central serous chorioretinopathy using artificial intelligence. Sci Rep. (2021) 11:20446. 10.1038/s41598-021-99977-4 34650220 PMC8516921

[82] Yoon J Han J Park JI Hwang JS Han JM Sohn J. Optical coherence tomography-based deep-learning model for detecting central serous chorioretinopathy. Sci Rep. (2020) 10:18852. 10.1038/s41598-020-75816-w 33139813 PMC7608618

[83] Kuo MT Hsu BW Lin YS Fang PC Yu HJ Chen A. Comparisons of deep learning algorithms for diagnosing bacterial keratitis *via* external eye photographs. Sci Rep. (2021) 11:24227. 10.1038/s41598-021-03572-6 34930952 PMC8688438

[84] Wang Z Zhong Y Yao M Ma Y Zhang W Li C. Automated segmentation of macular edema for the diagnosis of ocular disease using deep learning method. Sci Rep. (2021) 11:13392. 10.1038/s41598-021-92458-8 34183684 PMC8238965

[85] Li Z Guo C Nie D Lin D Cui T Zhu Y. Automated detection of retinal exudates and drusen in ultra-widefield fundus images based on deep learning. Eye (Lond). (2022) 36:1681–6. 10.1038/s41433-021-01715-7 34345030 PMC9307785

[86] Cabitza F Rasoini R Gensini GF. Unintended consequences of machine learning in medicine. JAMA. (2017) 318:517–8. 10.1001/jama.2017.7797 28727867

[87] Caruana R Lou Y Gehrke J Koch P Sturm M Elhadad N. Intelligible models for healthcare: predicting pneumonia risk and hospital 30-day readmission. In: Proceedings of the 21th ACM SIGKDD International Conference on Knowledge Discovery and Data Mining (KDD '15). New York, NY (2015) 1721–30.

